# Role and Regulation of Glutathione Metabolism in *Plasmodium falciparum*

**DOI:** 10.3390/molecules200610511

**Published:** 2015-06-08

**Authors:** Sylke Müller

**Affiliations:** Institute of Infection, Immunity and Inflammation, College of Medical, Veterinary and Life Sciences, University of Glasgow, 120 University Place, Glasgow G12 8TA, UK; E-Mail: sylke.muller@glasgow.ac.uk; Tel.: +44-(0)141-330-2383; Fax.: +44-(0)141-330-4600

**Keywords:** malaria, glutathione, drug discovery, drug target, redox metabolism, antioxidant

## Abstract

Malaria in humans is caused by one of five species of obligate intracellular protozoan parasites of the genus *Plasmodium. P. falciparum* causes the most severe disease and is responsible for 600,000 deaths annually, primarily in Sub-Saharan Africa. It has long been suggested that during their development, malaria parasites are exposed to environmental and metabolic stresses. One strategy to drug discovery was to increase these stresses by interfering with the parasites’ antioxidant and redox systems, which may be a valuable approach to disease intervention. *Plasmodium* possesses two redox systems—the thioredoxin and the glutathione system—with overlapping but also distinct functions. Glutathione is the most abundant low molecular weight redox active thiol in the parasites existing primarily in its reduced form representing an excellent thiol redox buffer. This allows for an efficient maintenance of the intracellular reducing environment of the parasite cytoplasm and its organelles. This review will highlight the mechanisms that are responsible for sustaining an adequate concentration of glutathione and maintaining its redox state in *Plasmodium.* It will provide a summary of the functions of the tripeptide and will discuss the potential of glutathione metabolism for drug discovery against human malaria parasites.

## 1. Introduction

Malaria in humans is caused by five species of protozoan parasites of the genus *Plasmodium*, namely *P. falciparum*, *P. vivax*, *P. ovale*, *P. malariae* and *P. knowlesi* (www.cdc.gov). The most severe form of the disease is caused by *P. falciparum* and about 90% of the 600,000 annual deaths are attributable to an infection with this parasite species in Sub-Saharan Africa, with children under the age of 5 and pregnant women being most vulnerable to the infection [1].

The parasites causing malaria have a complex life cycle, which depends on their sexual development in female *Anopheles* mosquitoes where the human infective transmission stages (the so-called sporozoites) are formed. When the mosquito bites a human, the infective sporozoites invade hepatocytes, where they undergo a first, non-symptomatic multiplication cycle by schizogony, before thousands of merozoites are released into the blood stream. Here, they infect red blood cells (RBC) and within 24 h, 48 h or 72 h (depending on the *Plasmodium* species) develop into up to 32 daughter merozoites. These egress from the host cells, infect fresh RBC and begin a new round of multiplication. At this stage of their cycle, the parasites can also develop into gametocytes, which are infective to mosquitoes—thus closing the cycle. The RBC stages of *Plasmodium* cause the symptoms of malaria and are those that most of the currently available chemotherapies act against. However, resistance against a lot of these drugs rapidly develops and spreads, and this alarming situation underpins the urgency for the development of new intervention strategies to fight the parasites and thus prevent or treat malaria, especially as an efficacious vaccine is still elusive.

Evidence suggests that the geographical distribution of RBC genetic traits such as the thalassemias, sickle cell anaemia and glucose-6-phosphate dehydrogenase deficiency (G6PDH-deficiency) correlate with reduced severity of malaria [2,3,4,5,6]. How these haemoglobinopathies interfere with the infection remains uncertain although some hypotheses suggest increased oxidative stress may play a pivotal role. The underlying mechanisms may involve the oxidative damage to the host RBC that occurs upon infection with *Plasmodium* and that these are exacerbated by the genetic traits [5,7,8]. The phenotype of *Plasmodium*-infected RBC harbouring one of the genetic traits resembles that displayed by senescent or pathologically damaged RBC [5] and this may result in a reduced “*Plasmodium*-infectivity” [9]. An infection with *Plasmodium* results in an increased level of reactive oxygen species in the infected RBC [10,11,12,13,14]. This leads to premature oxidative damage of the infected RBC, which ultimately affects membrane composition and protein conformations of the infected host cell and enhances the generation of lipid peroxides such as 4-hydroxynonenal. These lipid peroxides negatively affect RBC membrane integrity and impact on the host’s immune response *in vitro* and in infected individuals [15,16].

Nevertheless the parasites manage to thrive in this hostile environment presumably because they possess an extraordinary capacity to withstand the oxidative challenges that they encounter during their intra-erythrocytic growth [17,18]. Two redox systems help the parasites in this fight for survival. They possess functional and highly elaborate thioredoxin and glutathione redox systems in addition to other necessary components of an active antioxidant defence [18,19,20]. Both redox systems rely on a supply of NADPH required as reducing equivalents for their disulphide oxidoreductases thioredoxin and glutathione reductase (GR), respectively [18,20,21]. Usually, NADPH is provided by the pentose phosphate shunt (PPP) and this is also the case in *Plasmodium* [22,23,24,25]. In *P. falciparum* it was found that glucose-6-phosphate dehydrogenase and 6-phosphogluconolactonase form a bifunctional enzyme providing NADPH for downstream redox reactions [26]. Although this enzyme might supply the cytoplasm with sufficient reducing equivalents, it is likely that other NADPH-generating dehydrogenases present in the parasite’s organelles also contribute to the supply of NADPH. For instance, *Plasmodium* possess two NADP^+^-dependent glutamate dehydrogenases—one is cytosolic, while the other one is located in the plastid-like organelle called apicoplast [27,28]. In addition, the parasites contain a mitochondrial NADP^+^-dependent isocitrate dehydrogenase generating NADPH [29]. Thus, reducing power is also available in the apicoplast and mitochondrion—both organelles that also need to be protected against endogenous metabolic oxidative stress and therefore depend on an adequate supply of the redox co-factor. Over the past decades, the thioredoxin and glutathione redox systems have been extensively studied and their importance for parasite survival was evaluated. This review will focus on glutathione and its roles in parasite biology and it will discuss whether the pathways controlling glutathione homeostasis are suitable for drug discovery against malaria. In the following text, glutathione will be used when both reduced glutathione (GSH) and oxidized glutathione (glutathione disulphide, GSSG) are referred to.

## 2. Glutathione

The tripeptide glutathione (γ-glutamyl-cysteinyl-glycine) represents the major thiol redox buffer in almost all eukaryotes and thus is a determinant of the intracellular redox status. GSH is a co-factor for antioxidant and detoxification enzymes such as glutathione peroxidases (GPx) and glutathione S-transferases (GST) and its abundance and redox state impacts on the cell’s ability to defend itself against internal and external stresses. Glutathione normally is highly abundant (in mM concentrations) and as an enzyme co-factor and an antioxidant it is active in its reduced form as GSH, which is maintained by the NADPH-dependent GR [30]. The ratio of GSH to GSSG is usually high (>30:1) to ensure maintenance of the intracellular reducing environment. The only compartment with a more oxidising redox status is the endoplasmatic reticulum (ER), where the GSH/GSSG ratios have been reported to be up 1:1 [31]; this is necessary to ensure structural integrity of excreted or membrane proteins often containing multiple intra- or inter-molecular disulphide bonds. Therefore, GSH levels and GSH/GSSG ratio provide a suitable indicator of cellular redox homeostasis and oxidative stress [32,33,34].

This is also the case in the malaria parasite *Plasmodium*. These intracellular protozoan parasites possess a parasite-specific GR, which recycles GSSG and maintains thiol redox balance at the expense of NADPH [19,21,35,36,37]. The GSH/GSSG redox couple in intra-erythrocytic parasites has a highly reducing redox potential *E°_GSH_* of −314 mV comparable with the GSH/GSSG redox potential in other organisms [38]. *Pf*GR is found in both, the cytoplasm and the apicoplast of the parasites although they apparently only possess a single gene encoding the protein. It was shown that the dual location is attributable to alternative translational initiation directing the protein into cytoplasm and plastid organelle. This mechanism of guiding proteins encoded by a single gene into different cellular locations has been adapted by *Plasmodium* quite efficiently and it was found that thioredoxin reductase is also dually targeted through this mechanism to both cytoplasm and mitochondrion [39].

*Plasmodium* also possesses the two enzymes responsible for the *de novo* biosynthesis of GSH, γ-glutamylcysteine synthetase (γ-GCS) and glutathione synthetase (GS), respectively. The biosynthetic pathway for GSH establishes a constant source of the tripeptide in the parasites cytoplasm, as long as the amino acid precursors (glutamate, cysteine, and glycine) are available [40,41,42,43,44,45]. GSH biosynthesis is of particular importance in *Plasmodium-*infected RBC because the infected RBC “lose” or turnover glutathione rapidly and thus have to constantly replenish the tripeptide via their biosynthetic pathway [40,44,46]. It has also been suggested that uptake of GSH may contribute to sustain intracellular GSH levels; however, the significance of this source of GSH remains to be established [44,47,48]. The reason for the rapid glutathione turnover is unknown but one possible interpretation of this finding was provided by Ginsburg and colleagues suggesting that GSSG generated in the parasite may be excreted into the host RBC, which apparently has a decreased ability (or lost the ability altogether) to synthesise GSH *de novo* and this way provides the co-factor to its host cell, which contains GR to reduce GSSG back into GSH [49]. Another study provided evidence that it is primarily GSH that is excreted from the parasites [46].

*Plasmodium*-infected RBC encounter oxidative stress through the parasite’s metabolic activity and also the host’s immune system. *Plasmodium* incorporates and degrades the major host cell protein hemoglobin in its acidic digestive vacuole (DV) by a set of peptidases [50]. Heme (ferroprotoporphyrin IX containing Fe^2+^) and hemin/hematin (ferriprotoporphyrin IX containing Fe^3+^), highly redox active metabolic by-products of hemoglobin degradation, are released and the majority is biomineralised and sequestered in the form of hemozoin crystals within the DV to prevent downstream toxicity [51]. However, it has been suggested that appreciable amounts of heme may be released into other compartments of the parasite-infected RBC, where they interfere with protein and membrane integrity and affect the thiol redox balance [52]. One hypothesis is that hemin/hematin is removed by non-enzymatic degradation/peroxidation using GSH as observed *in vitro* [52,53]. This was recently confirmed and it was further demonstrated that hemin/hematin degradation was accelerated by the action of EXP1 (exported protein 1), a protein with GST activity located in the parasitophorous vacuole [54]. This enzyme activity may rely on GSH excreted from the parasites (which was demonstrated by Barrand and colleagues [46,55]) and this could be the explanation for the unusually fast turnover of the tripeptide observed in earlier studies [40,49]. In addition, the parasites possess a second, cytosolic GST, which until recently was considered to be the sole GST existing in *Plasmodium*. The protein shows classical GST activity using artificial substrates, is highly abundant in the parasite cytoplasm and was shown to bind and thus sequester hemin as ligandin; the enzyme also has some considerable antioxidant activity [56,57,58,59,60]. Curiously, the parasites contain no genuine GPx and the protein with a high sequence similarity to GPx from other organisms depends on thioredoxin as reducing cofactor [61]; further it needs to be mentioned that *Plasmodium* do not possess a gene encoding catalase [62]. Therefore, it is presumed that the multiple organelle-specific peroxiredoxin-linked detoxification systems of hydroperoxides provide the majority of the parasites’ antioxidant capacity [18,20,63,64].

Total glutathione levels have been determined in isolated *P. falciparum* and *P. falciparum-*infected RBC multiple times and depending on the method used they vary considerably between different studies and different *Plasmodium* species [40,41,44,45,49,65]. Total glutathione concentrations (comprising GSH and GSSG) in the range of 1 to 2 mM were reported in *Plasmodium*-infected RBCs and the parasites themselves [19,38,44,45], although in some studies lower concentrations were measured [41,65,66,67]. These discrepancies may be attributed to differences in sample preparations as well as detection methods; it was for instance found that concentrating the parasites or parasitized RBC using Percoll or MACS columns exerts oxidative stress on the cells leading to a reduction of GSH levels [45]. There are multiple ways of determining GSH levels in *Plasmodium*-infected RBCs or isolated parasites: (1) HPLC-based methods using derivatisation of thiols with fluorescent probes [44]; (2) colorimetric spectrometric assays using Ellman’s reagent [68] and (3) recently an *in vivo* method was developed using *P. falciparum* that express a human glutaredoxin 1 (hGrx) analogue linked to redox-active GFP (hGrx-roGFP), which allowed determining the GSH redox state in the parasites in real time without disrupting the parasite-host cell unit [38]. All detection methods conclusively demonstrate the GSH redox state is highly reducing in both host-parasite unit and isolated parasites, as expected, and moreover, the *in vivo* assay showed that the GSH/GSSG ratio and concentration is stable over time in unstressed parasites [38]. This is particularly interesting in light of the previously mentioned high turnover of GSH in the parasite suggesting an active biosynthesis of the tripeptide to compensate for its constant efflux from the parasite—be it as GSH, GS-X or GSSG [40,44,46,48]. Using hGrx-roGFP expression in *Plasmodium* now provides an excellent tool to monitor GSH redox balance in real time, assess the effect of pro-oxidants and other metabolic stressors and it should be possible to transfer the system to monitoring the redox balance in the different parasite organelles. This is an extremely exciting prospect and presents an excellent way to evaluate, for instance, the *in vivo* redox requirements of the protein trafficking machineries that are responsible for the transport of parasite proteins (virulence factors) into the host cell and its membranes operating in the *Plasmodium*-infected RBC or into the parasite’s organelles such as apicoplast, mitochondrion and ER.

## 3. The Functions of GSH in *Plasmodium*

**Figure 1:**
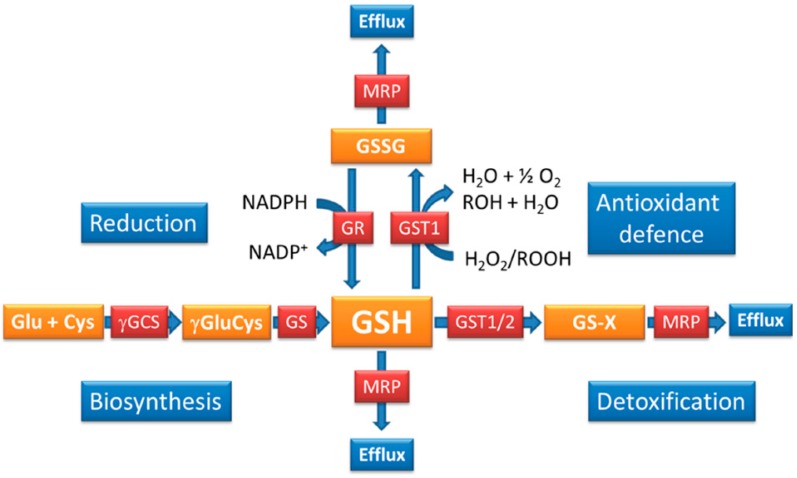
Regulation of GSH homeostasis in *P. falciparum.* Parasites contain GSH mainly in its reduced form [38] and its redox state is maintained by GR. GSSG and GSH are both effluxed from the parasites [46,49] to maintain GSH/GSSG redox balance. GSH is oxidised through antioxidant reactions primarily with GST1. Efflux, detoxification and antioxidant reactions lead to a constant loss of GSH, which is compensated for by GSH biosynthesis. Thus, efflux is counteracted by an active GSH biosynthesis, while oxidation of GSH is balanced through an active GR redox cycle.

*Plasmodium* possesses a number of GSH-dependent enzyme systems including GSTs, glyoxalases and glutaredoxins as well as a GPx-like protein [19,20,54,60,69]. Thus, GSH is important for detoxification and thiol-disulphide exchange reactions, however, its impact on the antioxidant capacity of the parasites is less well-defined especially as *Plasmodium* GPx is more likely to use thioredoxin as reducing co-factor rather than GSH [61]. It was shown that GST1, apart from detoxifying electrophiles and acting as a ligandin for hemin, also has GSH-dependent peroxidase activity and thus is an integral part of the parasite’s antioxidant machinery (Figure 1) [56,58]. A second GST activity (GST2), attributed to a protein previously called EXP1, was recently revealed and the protein is located in the parasitophorous vacuolar membrane surrounding the parasite. GST2 was shown to contribute to hemin/hematin-detoxification in a GSH-dependent manner as well as playing a role in the detoxification of the antimalarial artesunate [54]; its antioxidant capacity was not investigated. The glyoxalase system is another vital cellular component depending on a supply of reduced GSH and it was shown to be crucial for parasite survival [69]. Glyoxalases conjugate 2-oxoaldehydes such as methylglyoxal, a toxic metabolic by-product of glycolysis, to GSH and lead to the generation of non-toxic hydroxycarboxylic acids such as d-lactate, which are excreted from the cell. Apart from these pathways, the parasites possess numerous glutaredoxins and glutaredoxin-like proteins which require GSH as co-factors [70]. Together this indicates that the GSH system might not have the expected and often prominent role in providing an efficient antioxidant defence to *Plasmodium* but it seems to offer a powerful redox buffer that maintains intracellular redox homeostasis and is mainly involved in detoxification reactions through GSTs and glyoxalases and provides regulation of protein functions via thiol-disulphide exchange reactions as exemplified by the strong level of glutathionylation determined in *Plasmodium* proteins [71].

## 4. How is the GSH Redox Balance Regulated in *Plasmodium*?

Important insights into the mechanisms that contribute to regulating GSH levels as well as its redox state were obtained through specific inhibition of *de novo* GSH biosynthesis or inhibition of GR in *P. falciparum*-infected RBC [41,44]. More precise and targeted manipulation of these two key reactions became possible through the knockout of genes encoding γ-GCS and GR, respectively, in *P. berghei.* This offered insights into the potential importance of these two proteins in the animal model malaria species and allows to some extent to conclude also about the regulatory roles of these reactions for the maintenance of GSH redox balance in the human malaria parasites (Figure 1 and Table 1) [66,67,72].

**Table 1:** Comparison of *P. falciparum* and *P. berghei* protein and metabolite characteristics involved in glutathione metabolism.

	*P. falciparum*	*P. berghei*
Protein/Gene ID	Characteristics	Characteristics
Glutathione reductase (GR) Pf3D7_1419800.1; PBANKA_102340	Genetic validation: not done Chemical validation: essential in RBC stages [73] Location: cytoplasm and apicoplast [39]	Genetic validation: essential for sexual development [72] Chemical validation: not conclusive Location: not analysed
Thioredoxin reductase (TrxR)	Genetic validation: only possible in presence of	Genetic validation: not essential in any life cycle stage
Pf3D7_0923800.1; PBANKA_082470	expression plasmid [74]	
	Chemical validation: essential in RBC stages [75]	Chemical validation:
	Location: cytoplasm and mitochondrion [39]	Location: not investigated
Glutathione S-transferase 1 (GST1)	Genetic validation: not done	Genetic validation: not done
Pf3D7_1419300; PBANKA_102390	Chemical validation: essential in RBC stages [76]	Chemical validation: not conclusive [76,77]
	Location: predicted to be cytoplasmic	Location: not analysed
	No increase of activity with drug resistance [56]	Increased activity correlates with drug resistance [78,79,80]
	Has antioxidant activity [56]	Not assessed
Glutathione S-transferase 2 (EXP1)	Location: parasitophorous vacuolar membrane [54]	Protein was not studied with respect to GSH metabolism
Pf3D7_1121600; PBANKA_092670	Involved in heme and drug detoxification	
	Antioxidant activity not determined	
	Essentiality not determined	
γ-Glutamylcysteine synthetase (γGCS)	Genetic validation: impossible to KO gene [44]	Genetic validation: essential for sexual development [67]
Pf3D7_0918900; PBANKA_081980	Chemical validation: specific inhibition with BSO is	Chemical validation: specific inhibition does not affect
	lethal in RBC stages	parasite viability—not essential in RBC stages
	Location: cytoplasm	Location: not studied
GSH and drug resistance	GSH levels elevated in some drug resistant parasites [41,81]	GSH levels elevated in drug resistant parasites [78,79]
	GSH levels reduced in isogenic drug resistant	
	parasites [45]	

*P. falciparum* GR has been extensively studied over the past decades [20,82]. The gene was cloned, the recombinant protein expressed, characterised biochemically and its crystal structure was solved [35,83,84,85,86]. These studies allowed insights into the structural requirements of potential parasite-specific inhibitors and this has been taken forward in many studies since [73]. However, the recent gene deletion studies in *P. berghei*, a mouse malaria species that is frequently used as animal model for the human malaria species *P. falciparum*, revealed that GR is not essential for the survival of the intra-erythrocytic stages of *P. berghei* [66,72]. The knockout of *P. berghei gr* had little effect on parasite GSH levels and did not obviously affect intra-erythrocytic viability or parasite growth, providing strong evidence that GR in *P. berghei* is a redundant protein during intra-erythrocytic life and that its function is compensated for by alternative mechanisms, one being the thioredoxin redox system. This is reminiscent of *Saccharamyces cerevisiae* lacking *gr*, where the thioredoxin system provides an alternative reducing system that maintains the intracellular GSH/GSSG redox balance [87]. Apart from an alternative reducing system for GSSG, it is possible that the GSH/GSSG balance is sustained by efflux of GSSG and an increase of GSH *de novo* biosynthesis compensates for the loss of the tripeptide (Figure 1) [40,44,49]. This is feasible given that attempts to generate a double knockout mutant lacking both *gr* and γ*-gcs* were unsuccessful in *P. berghei* [72]*.* Another possible mechanism that could compensate for the loss of GR function is the uptake of GSH, but there is little evidence that GSH uptake into the parasite is sufficiently efficient to allow for the loss of the highly efficient GR activity; although this has not been experimental assessed in *P. berghei gr* deletion mutants, which would be highly informative [44,48]. The data suggest that it is difficult to deregulate the GSSG/GSH redox balance efficiently during intra-erythrocytic growth of the parasites, particularly in *P. berghei*. This may not be the case in *P. falciparum*, where so far no report about a deletion of the *gr* gene or the attempt thereof has been published (Table 1); in our lab a knockout of *Pfgr* was impossible to date (unpublished data). This implies that *Pf*GR may have a more pronounced function for the maintenance of the parasite’s redox homeostasis during their intra-erythrocytic growth than is the case in *P. berghei.*

The deletion of *gr* in *P. berghei* impacted however, on the progression of the parasites through their sexual cycle in the mosquito vector [66,72]. This part of the growth cycle was completely abrogated providing evidence that the loss of GR function in *P. berghei* cannot be replaced by the thioredoxin redox cycle during sexual development and that the two redox systems must have distinct roles during the parasites’ progression through these stages of their life cycle. This is further emphasised by the finding that thioredoxin reductase is not essential in any of the life cycle stages (including the sexual stages) of the *P. berghei* [72], showing that although there is some functional redundancy between the two redox systems during the erythrocytic cycle their individual roles must differ significantly in other parts of the parasite’s life cycle (Table 1). The precise reason for this selective effect of the *gr* deletion is unknown, but it has been speculated that GSH plays a pivotal role during the development of the *Plasmodium* in the mosquito, because the mosquito itself does not contain GR but relies on the reduction of GSSG by alternative redox systems [88]. Interestingly, a gene disruption or deletion of *thioredoxin reductase* in the human malaria species *P. falciparum* was impossible potentially highlighting differences in the redox biology of different *Plasmodium* species (Table 1) [74]. However, it cannot be excluded that the unsuccessful attempts to generate a *thioredoxin reductase* deletion in *P. falciparum* reflect technical limitations of the *P. falciparum in vitro* culture and knockout techniques used at the time of the study. One way of circumventing these limitations is the recent advent of conditional gene deletion systems in *P. falciparum* [89], which will allow revisiting this approach and elucidating to what extend the requirement for active redox systems during intra-erythrocytic development of *P. falciparum* and *P. berghei* differ and may represent distinct and parasite-specific metabolic adaptations.

Apart from redundancy between the two major redox systems involving GR and thioredoxin reductase, GSH levels are regulated through the biosynthetic pathway (Figure 1). GSH is synthesized *de novo* by two consecutive ATP-dependent enzymatic reactions catalysed by γ-glutamylcysteine synthetase (γGCS) and glutathione synthetase (GS) [32]. *Plasmodium* parasites possess both γGCS and GS [42,43]; both proteins are located in the parasite cytoplasm [45] similar to the situation in mammalian cells [90]. γGCS is considered the rate-limiting step of GSH biosynthesis while GS appears to be constitutively active without being post-translationally regulated [32]. Availability of GSH either through *de novo* biosynthesis regulated by γGCS or provided externally is essential in *S. cerevisiae*, *Schizosaccharamyces pombe*, *Candida albicans* as well as the protozoan parasite *Trypanosoma brucei* [91,92,93,94,95,96] and a knockout of γ*gcs* in mammals results in embryonic lethality [97]. These data emphasise the importance of GSH biosynthetic pathway for maintaining an adequate level of GSH in multiple eukaryotes and suggests that it may be a suitable target for intervention strategies should this also hold true for the *Plasmodium* parasites.

Mammalian γGCS has a catalytic and a regulatory subunit, which are distinct gene products Huang [98,99]. The enzymes from bacteria, fungi and the protozoan parasites *T. brucei* and *Plasmodium* consist only of the catalytic subunit [42,96,100,101,102,103], proposing that there are fundamental differences between the mammalian (host) proteins and those operating in protozoan parasites underpinning the potential for chemotherapeutic exploitation. In mammalian cells, the regulatory and catalytic subunits of γGCS form a heterodimer, which increases catalytic activity and decreases response to GSH feedback inhibition [104,105]. The relative abundance of the two protein subunits differs in a tissue-specific manner allowing a site/tissue-specific regulation of GSH production [106].

Apart from the post-translational regulation of γGCS, GSH biosynthesis depends on the satisfactory supply of its precursor amino acids l-glutamate, l-cysteine and l-glycine with l-cysteine generally considered the limiting component [107]. The availability of the three amino acids links GSH biosynthesis directly to nitrogen, carbon and sulphur metabolism and thus reflects the cell’s metabolic status. This is also the case in *Plasmodium* where metabolic re-modelling occurs many times in their developmental cycles in the two hosts. During the growth cycle in the RBC, the parasites obtain the majority of their amino acids through the digestion of hemoglobin. The only truly essential amino acid during this developmental stage of the parasites appears to be isoleucine while omitting other amino acids from the growth medium *in vitro* restricts growth but is not lethal to the parasites [108,109]. Genes encoding cystathionine β-synthase and cystathionine γ-lyase are lacking from the parasite genome [62] implying that they are unable to generate cysteine via transsulfuration from methionine and that they rely on cysteine/cystine uptake from their environment/host. Given that there is only a small number of cysteine residues (3) or methionine residues (5) present in the hemoglobin peptide chains restricting the availability of the sulphur-containing amino acid required for GSH biosynthesis, it is likely that an additional supply of sulphur-containing amino acids is required for optimal parasite growth. The availability of glutamine *in vitro* is high (the culture medium is usually supplemented with 2 mM glutamine) and *in vivo* its serum concentration is ~500 µM [110]. Recent targeted metabolomics studies of *Plasmodium* carbon metabolism revealed that during blood stage development, the parasites rely heavily on the supply of exogenous glutamine delivering the carbon skeleton that is fed into the mitochondrial tricarboxylic acid cycle as well as providing amino nitrogen for transamination reactions [111,112]. The glycine concentration in human serum is ~250 µM and the amino acid may also be provided via carbon or lipid metabolism in the parasites, similar to other eukaryotes [110,113,114]. It is therefore likely that the parasites depend on a supply of the precursor amino acids required for GSH biosynthesis largely from their host and this may vary with their dietary situation. Studies investigating the importance of GSH biosynthesis in the human malaria parasite *P. falciparum* suggest that the pathway is important if not essential for parasite survival. This was established using both chemical and genetic approaches (Table 1) [40,44]. The deletion of the γ*gcs* gene was impossible in *P. falciparum* suggesting that it is essential for the human malaria parasite and inhibition of GSH biosynthesis using the irreversible and specific inhibitor of γGCS buthionine sulfoximine (BSO) killed the parasites at µM concentrations [41,44]. However, the biosynthesis of GSH is not essential for the intra-erythrocytic development of the murine malaria species *P. berghei* [67] and accordingly BSO has no discernible effect on these parasites even at high concentrations [115] (Table 1). The knockout of γ*gcs* in *P. berghei* led to a reduction of total GSH levels and reduced growth rate of the mutant parasites in their mammalian host marginally. It was observed that mutant parasites preferably infected reticulocytes [67], which metabolically differ significantly from mature RBC [115].

The fact that a knockout of the γ*gcs* gene was possible in *P. berghei* but not in the human malaria parasite *P. falciparum* is reminiscent of the data obtained for a gene deletion of *P. falciparum* and *P. berghei thioredoxin reductase* genes mentioned above (Table 1) [44,67]. *Thioredoxin reductase* was shown to be non-essential throughout the entire life cycle of *P. berghei* [72], while it was impossible to knockout the gene in *P. falciparum* [74]. These diverse results support the hypothesis that the host environment (for instance the parasite’s host cell preference) may change and impact on parasite growth and survival rates and that therefore it is important to interrogate data from animal models carefully before drawing final conclusions as to the suitability of biological targets only validated in an animal model parasite. As outlined above, new conditional knockout systems are now becoming available to allow more robust assessment of drug targets in the human parasite *P. falciparum* experimentally. However, there are also important advantages of using the animal model parasite *P. berghei* to assess and validate potential drug target candidates in malaria parasites*.* The speed, ease and throughput with which the *P. berghei* system allows genetic manipulation and assessment of gene essentiality provides an enormous benefit [116] and the additional bonus of being able to assess the effects of gene knockouts on the growth and development of all life cycles stages of the parasites that are much less amenable to study with *P. falciparum* cannot be underestimated*.* In fact, using *P. berghei* it was demonstrated that the increased metabolic activity in the mitochondrion of sexual stages of the parasites is one reason for the essential role of GSH [67].

Other approaches that allowed insights into the regulatory mechanisms governing GSH/GSSG redox balance were obtained using the chemical inhibition of γGCS by BSO in *P. falciparum*-infected RBC. It was shown that *P. falciparum-*infected RBC turn over GSH much faster than the uninfected host cell [40]; this highlights the importance of the pathway for the parasites. This was further accentuated by the fact that inhibition of γGCS is lethal for *P. falciparum in vitro*, supporting that GSH biosynthesis is essential for parasite survival during the RBC cycle [44]. Overexpression of the γ*gcs* gene in *P. falciparum* affected GSH redox homeostasis and resulted in an adaptive decrease of GR protein levels and an increase in GSH/GSSG efflux. These data support the idea that the GSH redox balance is carefully guarded and adjusted by GR activity and GSH/GSSG efflux, if γGCS activity is elevated and presumably also when it is reduced [44,46,49]. These findings summarise that reduction of GSSG, biosynthesis of GSH and efflux of the tripeptide are primarily responsible for the regulation of intracellular GSH homeostasis (Figure 1).

Another mechanism that also might be involved and has been suggested to compensate for the loss of GSH biosynthesis is uptake of the tripeptide as has been shown in yeast [48,117]. However, the nature of the transporter is not clear and there are no obvious homologues of high-affinity GSH transporters belonging to the family of oligopeptide transporters (OPT) found in yeast, plants and bacteria in the *P. falciparum* genome [118]. However, a recent study has suggested that the DV chloroquine resistance transporter *Pf*CRT is involved in oligopeptide transport in *P. falciparum* and the transporter has also been implicated in the transport of GSH possibly providing the tripeptide ingested by the parasites into the DV to the parasites [45,119,120]; plant homologues of *Pf*CRT located in the chloroplast membrane were identified and shown to efficiently transport GSH [121].

## 5. Glutathione and Its Role in Drug Resistance

Using the redox active probe hGrx-roGFP a recent study showed that chloroquine, artemisinin derivatives, methylene blue, redox cyclers and pro-oxidants as well as BSO affect GSH redox balance in *P. falciparum* directly [38]*.* It was also demonstrated that the two *P. falciparum* strains 3D7 and Dd2 contain differential GSH redox capacities to respond to extracellular pro-oxidant challenges suggesting that the multidrug resistant parasites Dd2 have either elevated GSH concentrations or an elevated ability to maintain their GSH redox balance more efficiently than the drug sensitive parasites 3D7 [38,41]. This is in agreement with previous work showing that different parasite isolates contain differing levels of GSH and that these differences may correlate to some extent with their drug resistance phenotypes [38,41,45]. This hypothesis was particularly closely investigated for the antimalarial chloroquine and drug sensitive with drug resistant parasites were compared. The primary determinant of chloroquine resistance is associated with mutations in the *P. falciparum Pf*CRT [122]. It is generally accepted that the principal mode of chloroquine action relies on its accumulation in the parasite DV due to its basic character; in the acidic environment of the DV, chloroquine is protonated trapping the drug in the DV where it interferes with the biomineralisation of toxic heme into hemozoin [123,124]. The release of heme is thought to be lethal to the parasites presumably due to its toxicity [125,126]. Mutations of *Pf*CRT are associated with reduced accumulation of the drug in the DV and it was shown that the mutated transporter is responsible for the export of chloroquine from the DV. This was corroborated by *in vitro* studies showing that mutated *Pf*CRT facilitates transport of chloroquine [45,119]. However, levels of chloroquine resistance vary considerably between parasite isolates suggesting other secondary determining or modulating factors being involved in the mechanism of chloroquine resistance. Mutations in *Pf*Pgh1 transporter, a member of the MDR family of ATP transporters, and *Pf*MRP, a member of the multidrug resistance associated protein family, were shown to be secondary determinants of the level of chloroquine resistance [48,127,128,129,130] although the suggested correlation with mutations in *Pf*MRP and the transporter’s contribution to resistance against chloroquine is controversial [48,131].

In addition, varying concentrations of GSH were proposed to modulate the level of chloroquine resistance [41,45,48,49,52]. The association between elevated GSH levels and chloroquine resistance was convincingly shown in the animal model malaria species *P. berghei*. Increased GSH levels correlated with elevated GST activity, and reduced formation of hemozoin (presumably a higher detoxification of heme through the GSH-GST detoxification system) in the murine parasites (Table 1) [47,78,79,132]. For the human malaria parasite *P. falciparum* controversial data have been reported [45,133], which may reflect varying responses by the human malaria parasites to the changing capability of heme detoxification due to chloroquine action. However, upon treatment with chloroquine increased levels of hemin were found to accumulate in membranes isolated from *P. falciparum-*parasitized RBC, which correlated with parasite death [126] suggesting that formation of hemozoin is likely to be negatively affected upon chloroquine treatment. The hypothesis that elevated GSH levels increase resistance to chloroquine [41,126,134] was queried when the effect of GSH levels on chloroquine resistance was investigated in isogenic chloroquine sensitive and resistant *P. falciparum*. The study by Patzewitz and colleagues [45] showed that chloroquine resistant parasites (that only differ in their *crt* loci from the sensitive lines) had *reduced* levels of GSH rather than *elevated* GSH levels (Table 1) [45]. It was further demonstrated that mutant *Pf*CRT transports GSH likely into the DV where it may have a role in protecting parasites against the toxicity of free heme by degrading it, leading to an increased tolerance towards the drug [45,81,135,136,137,138]. Support for this theory was recently provided by a genetic metabolomics/quantitative trait locus analysis evaluating the metabolite composition of the *P. falciparum* progeny of a genetic cross of chloroquine resistant and chloroquine sensitive parasites [120]. This study provided evidence that the natural role of *Pf*CRT is linked to protein degradation in the DV and likely peptide transport across the DV membrane. It showed that mutations of *Pf*CRT led to peptide accumulation (presumably due to reduced transport from the DV into the parasite cytoplasm), elevated levels of resistance against chloroquine (chloroquine possibly affecting peptide transport from the DV by competing for their transport by mutated *Pf*CRT) and reduced fitness of the resistant parasites. The latter may be attributable to the reduced availability of peptides/amino acids for downstream metabolic and biosynthetic activities (including amino acids required for the synthesis of GSH) in the chloroquine resistant parasites [120].

Comparing parasite isolates/strains with different genetic backgrounds suggested that elevated GSH levels may provide protection against other antimalarials such as artemisinin and its derivatives [38]. The association of GSH may potentially be a contributing factor to the levels of resistance against this class of antimalarials; the major determinants for artemisinin-resistance in *P. falciparum* are however, mutations in the conserved kelch protein K13 (PF3D7_1343700) encoded on chromosome 13 [139,140,141]. These polymorphisms now provide the first molecular markers to detect artemisinin resistance and this will be of vital importance to prevent further spread of resistance against these extremely efficient antimalarials. Apart from its structural similarity to kelch-domain containing proteins, nothing is known about the function of *Pf*K13, but it has been speculated that it is involved in protein-protein interactions and the parasite’s redox signalling in analogy to kelch-like proteins such as Keap1 and Nrf2 in other organisms [142].

## 6. Glutathione Metabolism of *Plasmodium*—A Drug Target?

Interfering with *Plasmodium* GSH homeostasis may provide a means to interfere with parasite survival at different stages of their complex life cycle. The parasites maintain a highly reducing intracellular GSH/GSSG ratio [38] thanks to GR and presumably their ability to modulate GSH and GSSG levels through efflux pumps [46,48,49]. GR has been studied extensively as potential target for drug discovery and 1,4-naphthoquinones and other redox cyclers have been identified as potential chemical leads [73]. Based on structural features of the parasite protein, chemicals binding in the homodimer intersubunit cavity were also found to be promising drug leads inhibiting the recombinant enzyme with high efficiency as well as displaying antiparasitic activity *in vitro* [143]. One downside is however, that GR was shown to be not essential in the RBC stages of *P. berghei* and unfortunately there is no information available about its essentiality in *P. falciparum in vitro.* Given that other proteins involved in the maintenance of the human parasite’s redox balance such as thioredoxin reductase and γ-GCS appear to be essential for the survival of RBC stages of *P. falciparum* while they are dispensable for the murine malaria parasites, suggests that there is further need to fully assess the role of GR in *P. falciparum* before concluding that this protein is not a suitable target for intervention against RBC stages of human malaria*.* However, even if GR is not essential during RBC development, the protein appears to be a possible transmission blocking drug target (Table 1). Similarly, the rate limiting enzyme of GSH biosynthesis, γ-GCS, was found to be essential during sexual development of *P. berghei* although mutant parasites had reduced GSH levels and preferably infected reticulocytes during blood stage cycle suggesting a greater requirement for a supply of the tripeptide from their host and implying an imbalance of GSH redox homeostasis [67]. However, similar to GR, a knockout of the gene encoding γ-GCS in *P. falciparum* was impossible and using BSO, the need for an active GSH biosynthesis pathway during intra-erythrocytic development of the human malaria species was demonstrated (Table 1) [40,44]. A downside is, however, that chemical validation with BSO may not be entirely specific. It was suggested in a previous study on *T. brucei* γ-GCS that the drug may have off target effects [95]. Overall, the distinct findings from the studies using *P. berghei* and *P. falciparum* may reflect metabolic differences of the two malaria species and the essentiality of GR or γ-GCS should be investigated using the technical advances that are currently being made with conditional knockout systems (Table 1). Both gene deletions resulted in an interruption of the sexual development of *P. berghei* strongly suggesting that the GSSG reduction and GSH biosynthesis pathways may be suitable targets for transmission blocking agents. This goal to block malaria transmission is imperative to achieve eradication of the disease [144]. However, further experimental evidence is required to fully validate and assess the importance of the proteins of the human malaria species to conclude that the glutathione pathway of *Plasmodium* is suitable for future drug discovery.

It also needs to be assessed and validated whether the proposed targets are druggable. There is some doubt about the suitability of the disulphide oxidoreductase GR as the enzyme was assessed exhaustively as a drug target using multiple chemical compound screening and medicinal chemistry approaches and, so far, the outcomes have been modest and have not resulted in chemical leads that are taken forward into the drug development pipeline [20,73,82,145]. BSO on the other hand has been used as experimental tool to study the importance of GSH biosynthesis in cancer and also to chemically validate GSH biosynthesis in protozoan parasites [40,44,95,146] and the notion of reducing intracellular GSH levels in combination with other treatments has been considered a valid approach in anticancer chemotherapy. However, the inability to recombinantly express the *Plasmodium* γ-GCS and the uncertainty about its essential function in the human *Plasmodium* species has hindered progress to fully evaluating *Plasmodium* γGCS as a drug target. Overall, it remains for more robust and conclusive evidence to be generated to convince and engage the pharmaceutical industry in a drug discovery project with GSH metabolism of malaria parasites as a target.

## List of Abbreviations

BSO: buthionine sulfoximine
*Pf*CRT: *Plasmodium falciparum* chloroquine resistance transporter
DV: digestive vacuole
ER: endoplasmatic reticulum
EXP1: exported protein 1
γ-GCS: γ-glutamylcysteine synthetase
GSH: reduced glutathione
GSSG: glutathione disulphide
GPx: glutathione peroxidase
GST: glutathione *S*-transferase
GR: glutathione reductase
GS: glutathione synthetase
PPP: pentose phosphate shunt
RBC: red blood cells
